# Analytical and Clinical Validation of Solo‐Test Driver: A Targeted Amplicon‐Based NGS Test‐System for FFPE and cfDNA Analysis in Clinical Oncology Setting

**DOI:** 10.1002/jcla.70008

**Published:** 2025-03-08

**Authors:** Maxim Ivanov, Alexandra Lebedeva, Ekaterina Belova, Tatiana Grigoreva, Egor Veselovsky, Alexandra Kavun, Anastasiia Taraskina, Olesya Kuznetsova, Vladislav Nikulin, Laima Belyaeva, Daria Kravchuk, Tatyana Lisitsa, Alexey Barinov, Natalia Pospekhova, Saida Aliyarova, Ekaterina Khomenko, Alexey Tryakin, Irina Demidova, Anna Stroganova, Mikhail Fedyanin, Vladislav Mileyko

**Affiliations:** ^1^ OncoAtlas LLC Moscow Russia; ^2^ Sechenov First Moscow State Medical University Moscow Russia; ^3^ Lomonosov Moscow State University Moscow Russia; ^4^ Federal State Budgetary Institution N.N. Blokhin National Medical Research Center of Oncology Moscow Russian Federation; ^5^ State Budgetary Institution of Health Care of the City of Moscow “Moscow Multidisciplinary Clinical Center” “Kommunarka” of the Department of Health of the City of Moscow Moscow Russia; ^6^ Moscow City Oncology Hospital No 62 of the Moscow Health Department Istra Russia; ^7^ Federal State Budgetary Institution “National Medical and Surgical Center Named After N.I. Pirogov” of the Ministry of Health of the Russian Federation Moscow Russia

**Keywords:** amplicon panel, cfDNA, companion diagnostics, FFPE, NGS, tumor molecular profiling

## Abstract

**Background:**

Next‐generation sequencing (NGS) is increasingly integrated into cancer patient management, necessitating cost‐effective, reliable tests for companion diagnostics. We present the validation of the Solo‐test Driver panel, a custom NGS amplicon‐based tool for DNA analysis of 34 oncogenes, addressing key clinical needs.

**Methods:**

The panel's performance was validated for detecting SNVs, CNVs, and MSI. Analytical validation used 182 samples, while clinical validation involved 130 samples, both encompassing diverse tumor types and specimen formats.

**Results:**

The Solo‐test Driver panel has the potential to identify an additional 18.3%, 16.5%, and 8.7% of RAS+ colorectal, PIK3CA+ breast, and EGFR+ lung cancer patients, respectively, when compared to FDA‐approved PCR tests. Analytical validation demonstrated high intra‐lab robustness (coefficient of variation of coverage uniformity 6.4%) and high inter‐lab robustness (PCC of per‐amplicon coverage 0.82, 0.84, 0.9 for three different labs). Estimated in silico sensitivity was 100% for detecting clinically actionable SNVs at 250x, corresponding to only 60,000 read pairs per sample. In vitro mixing experiments determined LoD starting from 3.3% VAF. Estimated in silico LoD ranged from 0.5% to 5% at 1000× (1% to 5% at 650x). Clinical validation demonstrated PPA/NPA of 100%/80%, 95%/100%, and 100%/100% for detecting MSI, SNVs, and CNVs, respectively.

**Conclusions:**

The Solo‐test Driver panel offers a reliable, cost‐effective solution for detecting somatic alterations and genomic signatures, making it suitable for routine companion diagnostics in solid tumors.

## Graphical abstract

With the growing demand for compact next‐generation sequencing (NGS) test systems tailored to meet key needs in oncology, the Solo‐test Driver panel serves as a valuable tool for companion diagnostics of solid tumors, demonstrating high reliability, sensitivity, and specificity in line with established guidelines. In this work, we outline the framework used to validate panels like the Solo‐test Driver panel.

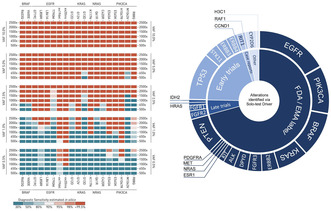

## 1 Introduction

In recent years, next‐generation sequencing (NGS) methods have become widespread, significantly increasing the volume of analyzed data and opening up opportunities for targeted medicine in precision oncology [1]. NGS is being used in a variety of molecular oncology applications, such as sequencing entire tumor genomes or using targeted diagnostic gene panels. Compared to traditional molecular diagnostic methods such as PCR, IHC, or FISH, NGS allows for the heterogeneity and genetic complexity of many tumors to be taken into account and is a more efficient method for obtaining a comprehensive tumor molecular profile. The number of FDA‐approved molecularly matched indications is increasing every year, as is the number of biomarker‐matched approved targeted drugs. Thus, the current availability and cost‐effectiveness of NGS and the improvement of bioinformatic data analysis programs have enabled the implementation of high‐throughput, sensitive, and accurate tumor profiling [2, 3, 4].

In Russia, 0.58 million new cancer cases were recorded in 2021 [5]. Only a small number of NGS panels for breast cancer, ovarian cancer, pancreas, and prostate cancer are currently registered; thus, most molecular genetic tests are performed using conventional methods. However, for driver somatic mutations in such common cancer types, such as colorectal cancer (12.2%), breast cancer (12.1%), lung cancer (9.7%), gastric cancer (5.5%), bladder cancer (2.7%), melanoma (2%), and bile duct cancer (2.2%) there is no available panel solution in the country. In modern clinical practice, the presence of driver mutations is detected using PCR, which allows to identify only the most common variants, or foreign multigene panels that are inaccessible to most patients due to high cost and require accurate interpretation of the results.

Herein we describe the results of analytical and clinical validation studies of a custom NGS‐based panel that includes analysis of small variants (SNVs, MNVs and up to 40 b.p. indels), whole gene deletions/amplifications (CNVs) and up to 71 STR regions for MSI analysis in a clinical oncology setting, for potential implementation in a community pathology and/or molecular diagnostics laboratory, to advance the detection rate of clinically relevant biomarkers and thus benefit oncology patient management and overall cancer care in the region. We developed an amplicon‐based test system for molecular alteration detection, designated Solo test Driver, with a focus on 34 oncogenes and 4 pharmacogenes, and evaluated the detection performance in FFPE and cfDNA specimens using the NGS platform based on sequencing by synthesis technology.

## 2 Materials and Methods

### 2.1 Panel Design

The panel design includes selected hotspots, whole exons, or complete coding regions of common genes that are known predictive, prognostic, and/or diagnostic biomarkers in the most frequently observed solid tumors. The panel includes the following genes: AKT1 (AKT serine/threonine kinase 1), AKT2 (AKT serine/threonine kinase 2), AKT3 (AKT serine/threonine kinase 2), ALK (ALK receptor tyrosine kinase), ARAF (A‐Raf proto‐oncogene, serine/threonine kinase), BRAF (B‐Raf proto‐oncogene, serine/threonine kinase), CYP2D6 (cytochrome P450 family 2 subfamily D member 6 (gene/pseudogene)), DPYD (dihydropyrimidine dehydrogenase), EGFR (epidermal growth factor receptor), ERBB2 (erb‐b2 receptor tyrosine kinase 2), ERBB3 (erb‐b2 receptor tyrosine kinase 3), ERBB4 (erb‐b2 receptor tyrosine kinase 4), ESR1 (estrogen receptor 1), FGFR1 (fibroblast growth factor receptor 1), FGFR2 (fibroblast growth factor receptor 2), FGFR3 (fibroblast growth factor receptor 3), FGFR4 (fibroblast growth factor receptor 4), G6PD (glucose‐6‐phosphate dehydrogenase), H3‐3A (H3.3 histone A), H3C2 (H3 clustered histone 2), H3C3 (H3 clustered histone 3), HRAS (HRas proto‐oncogene, GTPase), IDH1 (isocitrate dehydrogenase (NADP(+)) 1), IDH2 (isocitrate dehydrogenase (NADP(+)) 2), KIT (KIT proto‐oncogene, receptor tyrosine kinase), KRAS (Kirsten rat sarcoma viral oncogene), MET (MET proto‐oncogene, receptor tyrosine kinase), NRAS (NRAS proto‐oncogene, GTPase), PDGFRA (platelet derived growth factor receptor alpha), PIK3CA (phosphatidylinositol‐4,5‐bisphosphate 3‐kinase catalytic subunit alpha), PTEN (phosphatase and tensin homolog), RAC1 (Rac family small GTPase 1), RAF1 (Raf‐1 proto‐oncogene, serine/threonine kinase), RIT1 (Ras like without CAAX 1), ROS1 (ROS proto‐oncogene 1, receptor tyrosine kinase), STK11 (serine/threonine kinase 11), TERT (telomerase reverse transcriptase), TP53 (tumor protein p53), and UGT1A1 (UDP glucuronosyltransferase family 1 member A1). The gene‐therapy associations are summarized in Table S1. Additionally, the panel covers 71 STR loci to detect MSI and pharmacogenes associated with the toxicity or efficacy of treatments used in oncology by analyzing 10 high‐evidence SNPs according to PharmGKB. Also, the panel was designed to detect CNV in the following genes: *EGFR*, *ERBB2*, *ERBB3*, *ERBB4*, *FGFR1*, *FGFR2*, *FGFR3*, *FGFR4*, *MET*, and *PTEN*.

### 2.2 Tissue and DNA Samples

FFPE samples were retrospectively obtained from FSBI “National Medical Research Center of Oncology named after. N.N. Blokhin” of the Ministry of Health of Russia, MMKTs “Kommunarka” DZM, Moscow City Oncology Hospital No. 62. Plasma samples were retrospectively collected from MMKTs “Kommunarka” DZM. The study was approved by Sechenov University IRB declaring a waiver of informed consent and was conducted in accordance with principles claimed in the Declaration of Helsinki. DNA extraction from FFPE and plasma samples was done as previously described [6, 7]. A pathomorphological study was performed for each FFPE tissue sample before DNA isolation in order to select the most appropriate block for molecular genetic studies. The total number of tumor cells and their distribution in the sample, the presence of necrosis foci and the assessment of preanalytical disorders (mistakes/errors) in case of their presence were evaluated. DNA concentration was measured with fluorometer Fluo‐200 (AllSheng, China) using a Qubit ds DNA HS assay.

### 2.3 Library Preparation and Sequencing

Libraries were prepared using a custom Solo test Driver panel by target enrichment technology, based on multiplex PCR with two pools of primers. Amplicons were then partially digested and ligated to universal adapters, purified on magnetic beads, and amplified with index primers. The index primers encode individual 8‐base indices as well as an adaptor sequence, which can then bind to the flow cell (Illumina or GeneMined platform) through the adaptor sequence and be identified through sequencing of its unique index combination.

The quality control of the analyte included an assessment of the concentration of isolated DNA (at least 0.5 ng/μL), as well as an assessment of the concentration of DNA libraries (at least 0.6 ng/μL, which corresponds to 4 nmol with an average library length of 267 b.p.). In the case of low DNA concentration (from 0.5 to 1.5 ng/μL), the number of 1 PCR cycles increased to 21. In the case of a low concentration of the library (from 0.3 to 0.6 ng/μL), the possibility of further sequencing was determined privately (taking into account the concentration of DNA, as well as the percentage of tumor cells and the proportion of necrosis determined by the results of a pathomorphological examination of the initial sample). The target number of readings per sample with a low concentration of the library increased to at least 2.5million, which corresponds to an average coverage of 5000×. Library concentrations were measured by Fluo‐200 fluorometer (AllSheng, China) using dsDNA HS Assay Kit (Thermo Fisher Scientific).

Libraries were sequenced on a Genolab M (GeneMind) and FASTASeq300 (GeneMind) at 2 × 150 bp read length.

### 2.4 Bioinformatics Pipelines and Data Analysis

NGS data processing was done via “Solo AVES” web‐based platform tailored for the analysis of sequencing data generated by Solo test Driver and ABC Plus NGS‐based panels [8]. In brief, BWA [9] was used to align sequencing reads to the human genome GRCh37.p13 assembly. SNVs, MNVs, and indels calling pipeline included: (1) candidate gene variants calling with SiNVICT [10], STRELKA [11], and Mutect 2 [12]; (2) joining intersecting variations with in‐house algorithms; (3) collection of additional technical parameters of calling with Samtools and SGA [13]; (4) estimating variant calling probability versus reference database of health tissue sequencing with in‐house algorithm modeling site‐specific noise level with Poisson‐beta distribution; (5) final filtration of artifacts with an ML‐based model incorporating per‐variant technical parameters collected in previous steps along with prior probability of variant detection in specific tumor type. Interpretation of variants was done in two steps: (1) identifying the biological effect of variants via categorizing variants in oncogenes into activating (gain‐of‐function) or undefined via in‐house database of activating variants and further categorizing undefined variants in oncogenes and all variants in tumor suppressor genes into damaging, VUS, or neutral employing in‐house algorithm following ACMG recommendations; (2) clinical interpretation of specific gene variants identified in specific tumor type based on their biological effect via collation versus in‐house database based on NCCN, ASCO, and ESMO guidelines. CNV calling was done employing multifactor data normalization using reference database of health tissue sequencing. MSI analysis was done via estimating the sum of prevalences of k‐mers corresponding to shortened by 2 b.p. and longer as compared to references STR sequences. Cancer type nomenclature in the analysis analysis and throughout the manuscript is based on OncoTree codes [14].

### 2.5 Сomparison With Orthogonal Tests

For *in silico* screening of the MSK‐IMPACT dataset, the following PCR‐based diagnostic tests were selected: Therascreen KRAS RGQ PCR Kit (FDA cleared, Qiagen), Cobas KRAS Mutation Test for KRAS (FDA cleared, Roche Diagnostics), KRAS Mutation Test v2 (Roche Life Sciences) for *KRAS*; BRAF/NRAS Mutation Test (Roche Life Sciences) and Idylla NRAS‐BRAF Mutation Test (Biocartis) for *NRAS*; Therascreen PIK3CA RGQ PCR Kit (FDA cleared, Qiagen), Cobas PIK3CA Mutation Test (cobas PIK3CA Mutation Test, Roche Diagnostics), High Resolution Melting PCR (nonspecific real‐time PCR assay described previously [15]) for *PIK3CA*; Therascreen EGFR RGQ PCR Kit (FDA cleared, Qiagen), Cobas EGFR Mutation Test v2 (FDA cleared, Roche Diagnostics), and Idylla EGFR Mutation Test (Biocartis) for *EGFR*.

### 2.6 Concordance Analysis

The detection of genomic alterations (SNV, CNV) and MSI by the Solo test Driver panel was compared to the results of reference methods (gold standard). The externally validated OncoReveal Dx Lung and Colon Cancer (Pillar Biosciences, MA, USA) assay was used as a reference for SNV, CNV, and indel. For some COAD samples, PCR was used as a reference method for *KRAS*, *NRAS*, and *BRAF* SNV. For MSI analysis, conventional orthogonal methods were used, and samples were tested using both 5‐loci PCR assay and 4‐antibody immunohistochemical (IHC) staining. Samples were considered MSI‐positive (MSI+) if PCR and IHC both agreed on positive results. Consistently, samples were considered negative if the results of both methods were negative.

## 3 Results

### 3.1 Analytical Validation

A total of 39 genes covering 53.8 kb of sequence length with a total of 474 amplicons were included in the Solo test Driver panel. The panel covers 93.6% of 890 ESCAT I‐III (including 495 SNV and 338 indel) known clinically significant alterations in the main clinically significant genes (*ALK*, *BRAF*, *EGFR*, *ERBB2*, *ERBB3*, *ERBB4*, *ESR1*, *FGFR2*, *FGFR3*, *IDH1*, *KIT*, *KRAS*, *MET*, *NRAS*, *PDGFRA*, and *PIK3CA*). Additionally, 96% of 144 ESCAT I for six most commonly mutated genes that are tested for standard of care therapies (*EGFR*, *KRAS*, *NRAS*, *BRAF*, *PIK3CA*, and *ERBB2*) are covered by the panel (Table S1).

Next, we analyzed the coverage of known hotspots to assess the clinical utility of the panel. Using the data from the CancerHotspots project [16, 17], we evaluated the number of total alleles identified in solid tumors that would have been covered or missed by the panel. A total of 18,580 (98.5%) mutant alleles in genes included in the panel would have been detected, and 288 (1.5%) missed. When adjusted for standard of care (ESCAT I‐II) genes (i.e., *PIK3CA*, *ESR1*, and *ERBB2* for breast cancer; *KRAS*, *NRAS*, and *BRAF* for colon cancer, etc.), 37 (3.6%), 0 (0%), 1 (0.2%), and 0 (0%) of mutant alleles would have been negative among breast, bowel, lung, and skin cancer samples, respectively. Notably, 100% of all mutant alleles occurring in biliary tract, bladder, and CNS cancer samples would have been detected by the panel.

Next, we performed *in silico* estimation of additional information yield produced by the Solo test Driver panel compared to orthogonal PCR‐based molecular diagnostic tests based on coverage of clinically significant mutations according to test‐system design. For this purpose, we performed screening of patient data from the MSK‐IMPACT study [18] for clinically significant mutations employing different test systems (see Section 2). We selected clinically significant mutations in *KRAS*, *NRAS*, *PIK3CA*, and *EGFR* genes routinely tested for somatic mutation in real‐world practice. Then we selected patients from the MSK‐IMPACT dataset with cancer types in which testing of the genes listed above is required according to NCCN guidelines. A total of 3795 patients were included in the analysis, of which 982 had colorectal cancer with 421 *KRAS*‐positive cases and 36 *NRAS*‐positive cases, 1246 had breast cancer with 410 *PIK3CA*‐positive cases, and 1567 had non‐small cell lung cancer with 300 *EGFR*‐positive cases. Analysis showed that the Solo test Driver would allow additional detection of up to 65 (18.3%) *RAS*‐positive patients compared to FDA‐approved PCR tests. For the *PIK3CA* mutation, 58 (16.5%) patients would be additionally identified as compared to FDA‐approved test and from 21 (5.4%) to 62 (17.8%) as compared to commonly used PCR/HRM tests. Finally, for the *EGFR* mutation, additional information yield made up from 15 (5.3%) to 24 (8.7%) patients for FDA‐approved test and 16 (5.7%) patients for the commonly used PCR test.

**FIGURE 1:**
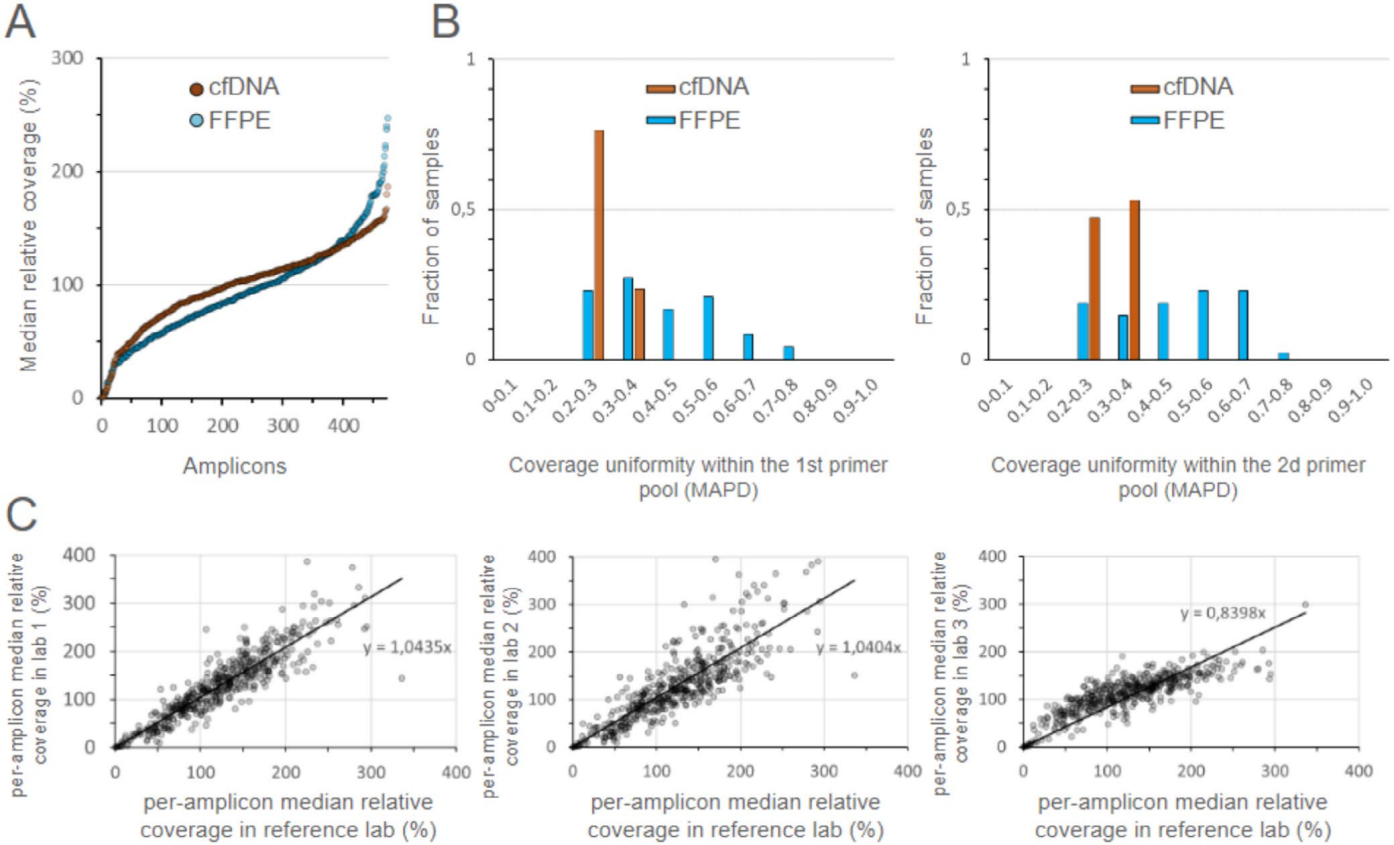
Uniformity of coverage distribution of FFPE and cfDNA samples by MAPD values for the first (A) and second (B) pools as well as inter‐lab robustness analysis (C).

To assess the repeatability of control metrics of data quality and coverage uniformity of target regions, 58 FFPE samples, 37 cfDNA samples, and 25 whole blood (WB) samples were sequenced in the single reference lab satisfying minimum coverage requirements (650× for FFPE, 1500× for cfDNA, and 150× WB; coverage coefficient of variation was 0.26, 0.24, and 0.21, respectively). The minimum on‐target fraction was 87% (mean value was 95% [95% CI, 94.6%–95.5%]) denoting a low level of off‐target amplification. Coverage uniformity was estimated via MAPD metrics [19]. The median in‐pool MAPD was 0.37 (mean values were 0.43 ± 0.13 for FFPE and 0.35 ± 0.09 for cfDNA, respectively; Figure 1B) with the second pool slightly outperforming the first one for both types of samples (average MAPD ratio was 1.11 [95% CI, 1.07–1.15] reaching 1.5 and higher for 3.1% of samples) and cfDNA samples demonstrating better coverage uniformity (Figure 1A). Mean e‐score values were 0.78 ± 0.05 for FFPE and 0.82 ± 0.05 for cfDNA, respectively. To assess the reproducibility of data quality, a total of 62 FFPE samples were sequenced in the reference lab and in three different labs which were supplied with the Solo test Driver test system, including the library preparation kit and data analysis software. Data quality was estimated in terms of per‐amplicon coverage relative to the average coverage depth of a single sample, which was averaged across all samples sequenced within a single lab and with results from the reference lab (Figure 1C). Pearson correlation coefficients were 0.82, 0.84, and 0.9.

Panel amplicon dropouts (amplicons with coverage less than 5% of the average) were also analyzed. Among FFPE samples, 7 systematic (more than in 10% of samples) amplicon dropouts were noticed, including those located in the *ERBB2*, *AKT3, FGFR1*, *TP53*, *RIT1*, and *EGFR* genes. Excluding systematic ones, there were 12 amplicon dropouts, and 8 amplicons dropped out more than once. For cfDNA samples, 6 systematic amplicon dropouts located in *ERBB2*, *AKT3*, *FGFR1*, *TP53*, and *STK11* genes and 13 non‐systematic ones were observed. Four systematic and four non‐systematic amplicon dropouts were the same for both sample types.

Clinical significance of amplicon dropouts was analyzed with EphaGen allowing to predict *in silico* sensitivity of selected sequencing dataset to detect selected range of mutations. Despite observed amplicon dropouts, predicted *in silico* sensitivity of detecting hotspot mutations [20] was 100% for all sequenced samples largely due to sufficient coverage and amplicon intersection. Furthermore, predicted *in silico* sensitivity was estimated separately (denoted further as single site sensitivity) for 14,784 clinically significant mutations with the highest (across diverse cancer types) level of evidence ESCAT I‐III. As a result, a single‐site sensitivity drop in any sequenced sample from 100% was noted only for 86 (0.58%) mutations (19 (0.36%) for ESCAT I; 45 (1.14%) for ESCAT II; 22 (0.39%) for ESCAT III).

**FIGURE 2:**
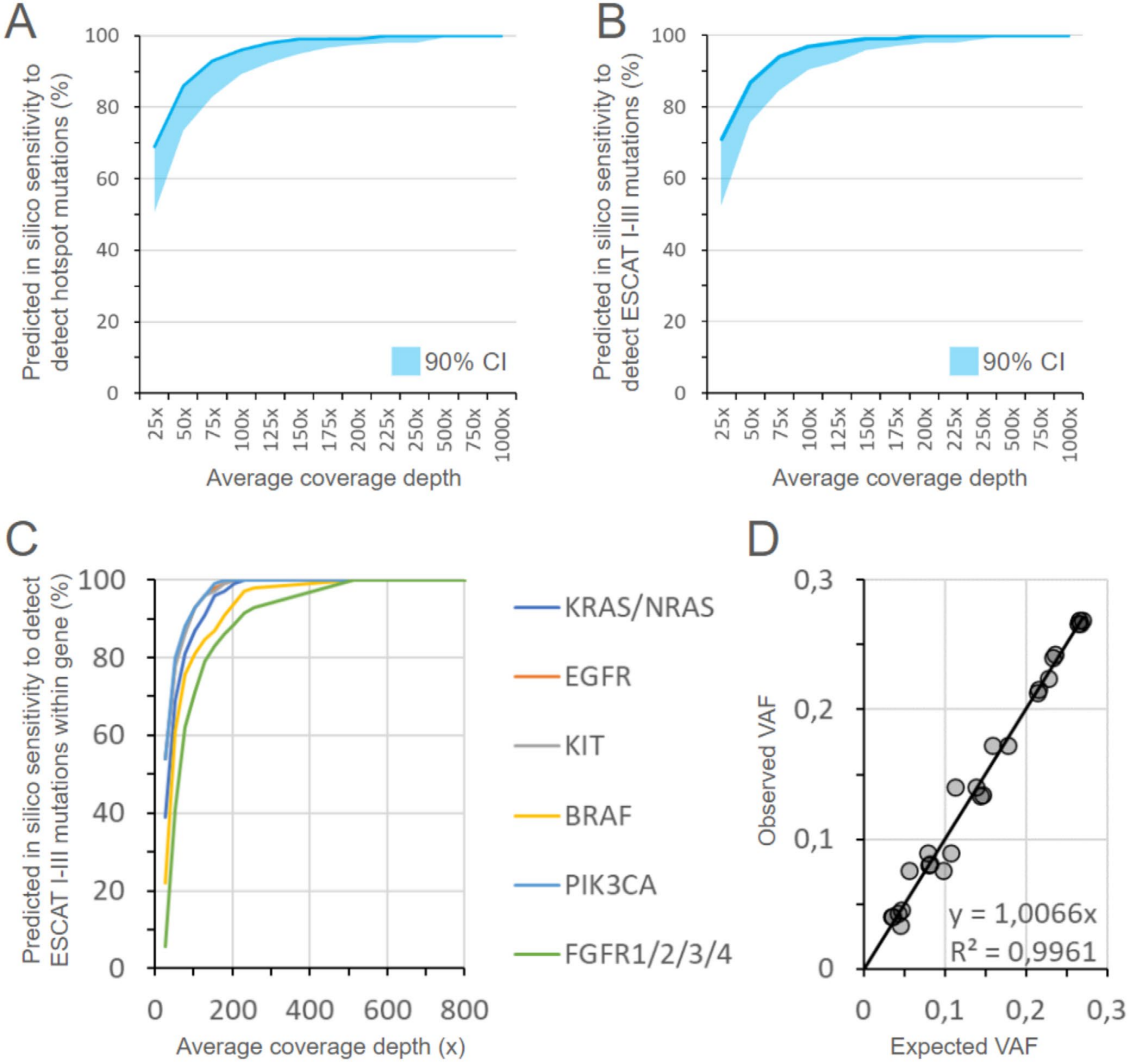
Predicted *in silico* sensitivity of the Solo test Driver panel for detecting hotspot mutations in 34 oncogenes (A), clinically significant (ESCAT I‐III) mutations (B) and per‐gene clinically significant (ESCAT I‐III) mutations (C) based on FFPE sequencing data demonstrates the 100% sensitivity of the panel to detect somatic mutations starting from 650x per‐dataset average coverage depth. Additionally, to validate the ability to detect subclonal mutations as well as validate the repeatability of VAF estimation, mixed in vitro DNA extracted from FFPE samples was used to generate a total of 26 somatic alleles in various VAF ranges (D) to correlate expected based on mixing proportions VAF versus observed based on mixed samples sequencing.

We also performed downsampling experiments with coverage decrease of 50%; 60%; 70%; 80%, and 90%–99%. The coverage of 230x was sufficient to achieve the hot spot mutation detection sensitivity of 100% (10th percentile was 98%; Figure 2A), and 250x for clinically actionable variants detection sensitivity of 100% (10th percentile was 99%; Figure 2B). For *KRAS*, *NRAS*, *EGFR*, *KIT*, and *PIK3CA* mutations, single site sensitivity reached 100% with a 200x coverage; for *BRAF* and *FGFR1*/*2*/*3*/*4* with a 500x coverage (Figure 2C).

Worth noting, covered regions included the *TERT* promoter region. Two amplicons located in *TERT* systematically dropped out in FFPE and cfDNA samples. Mean single site sensitivity was 98% [95% CI, 97.99%–98.93%].

The analytical sensitivity of VAF evaluation was calculated using in vitro mixed samples in prespecified proportions to obtain samples with a wide range of VAF mutations. DNA analytes extracted from FFPE different samples with a total of mutations (*EGFR*, *BRAF*, *PIK3CA*, *IDH1*, and *TP53*) previously confirmed by the Solo test Driver panel were mixed with DNA analytes extracted from FFPE samples previously sequenced with the Solo test Driver with no somatic mutations detected. Mixing proportions ranged from 1:2 to 1:25 to generate a total of 26 somatic alleles for the final analysis with expected VAF varying from 3.3% to 26.9%. As a result, the assay captured all somatic alleles expected to be observed in mixed samples (Figure 2D). A 0.99 coefficient of correlation (r‐squared) was extrapolated with a *p*‐value of 4.93 × 10^−23^ between expected variant allele frequencies (VAF) from the positive control and those reported by the assay. VAFs ranged from 27% to as low as 3.4%, showing the assay's high analytical sensitivity.

**FIGURE 3:**
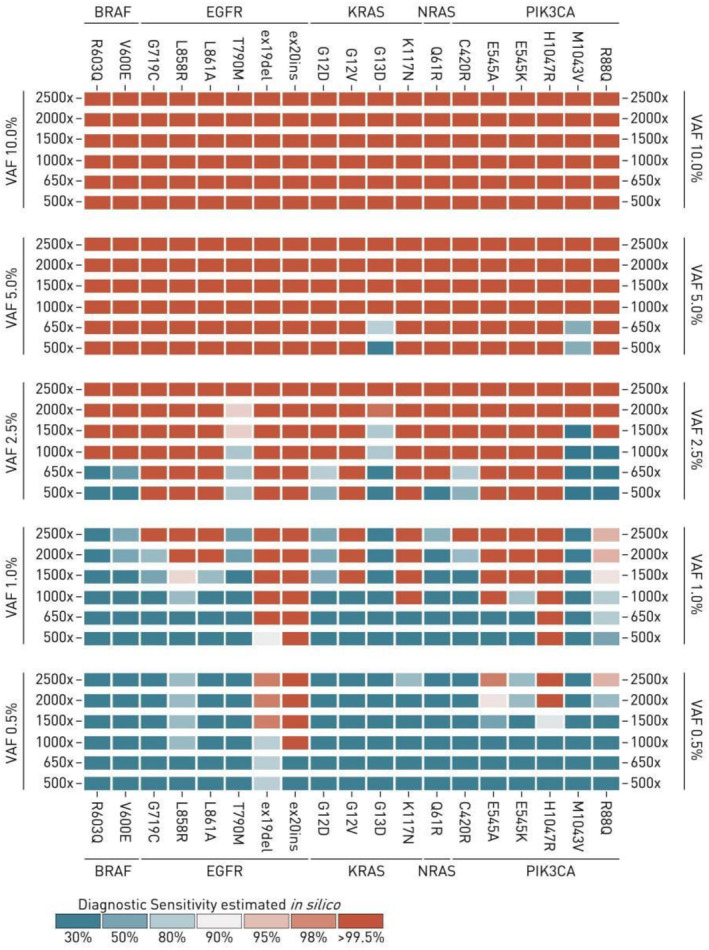
Mixed *in silico* datasets were used for the analysis of per‐mutation analytical sensitivity (limit of detection) of Solo‐test Driver panel at various per‐dataset coverage depth and various allele frequencies of target mutation. At least 100 datasets were generated for each combination of target mutation, allele frequency of target mutation and coverage depth of target mutation site and were used for calling target mutation. As a result, sensitivity could be estimated as a percentage of datasets in which target mutation could be successfully called.

Finally, we performed *in silico* mixing experiments to estimate analytical sensitivity (limit of detection) of the sequencing datasets generated by the Solo test Driver panel. For this, 39 FFPE samples confirmed to have at least single somatic mutation (a total of 23 unique alleles) were selected. Sequencing reads supporting somatic mutations were pulled out from sequencing datasets and mixed with one of the 10 sequencing datasets with no mutations detected with prespecified proportions to generate a set of prespecified counts of read pairs to generate a read pairs. Read pairs from the original dataset with confirmed mutations and the dataset with no mutations were selected randomly. The experiment was repeated at least 100 times for each combination of: (1) original FFPE sample with target somatic mutation expected to be detected in a mixed sample; (2) expected variant allele frequency of the target mutation; (3) coverage of the target mutation site. As a result, a total of 100,000 mixed *in silico* datasets were generated and used for calling target mutations. Sensitivity of detecting target mutations at specific allele frequency and coverage depth was estimated as frequency of successful calling (Figure 3). This demonstrated the ability of the Solo test Driver panel to detect *EGFR* exon 19 deletions with 100% sensitivity starting from 1% VAF and 650x coverage. *EGFR* exon 20 insertions could be called with 100% sensitivity starting from 0.5% VAF at 1000x coverage and 1% at 500x coverage. Any mutation could be called with sensitivity of 100% starting from 5% VAF at 1000x and higher.

### 3.2 Clinical Validation

**FIGURE 4:**
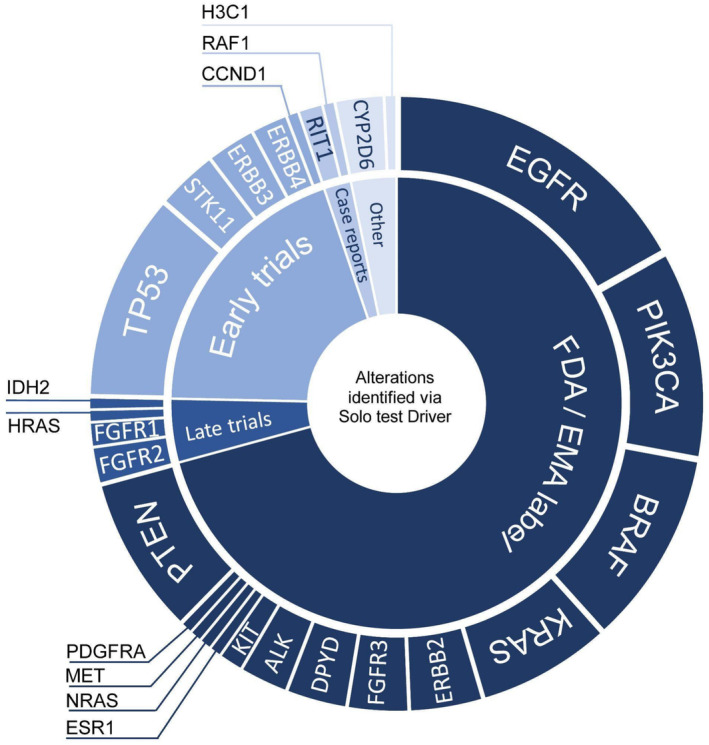
Predictive biomarkers identified in our study for therapy response in solid tumors according to the stage of development of associated molecularly matched therapy. Non‐standard of care pharmacogenomic and any diagnostic biomarkers were included in the “other” section. EMA, European Medicines Agency; FDA, US Food & Drug Administration.

The detection of alterations (SNV, CNV, and indel) was compared to the externally validated NGS assay (see Section 2). The spectrum of identified alterations is provided in Figure 4. The concordance analysis between Solo test Driver and Pillar CDx was performed on 46 FFPE samples (41—LUAD, 5—COAD) for 46 patients. Concordance analysis was performed in relation to clinically relevant alterations in ESCATI‐II level genes in LUAD (*EGFR*, *KRAS*, *MET*, *BRAF*, *ERBB2*, as well as *FGFR1* and *FGFR2*) and COAD (*KRAS*, *BRAF*, and *ERBB2*) [21]. A summary of Positive Percent Agreement (PPA), Negative Percent Agreement (NPA) and corresponding 95% two‐sided exact confidence intervals (CI) is provided in Table 1 below.

**TABLE 1:** Diagnostic accuracy of Solo test Driver for short variants (SNV, indel), CNV and MSI.

	Samples (*N*)	Gold standard	TP	TN	FP	FN	PPA [95% CI]	NPA [95% CI]
MSI	18	PCR/IHC	13	4	0	1	100% [NA]	80% [38–97]
All LUAD samples (excluding MSI)	41	NGS	29	12	1	0	97% [81–100]	100% [NA]
All COAD samples (excluding MSI)	13	PCR/NGS	11	1	1	0	91% [73–98]	100% [NA]
All COAD samples (including MSI)	20	PCR/NGS	18	1	0	1	100% [NA]	50% [13–87]
*EGFR* SNV in LUAD	41	NGS	13	29	0	0	100% [NA]	100% [NA]
*EGFR* indel in LUAD	41	NGS	12	29	0	0	100% [NA]	100% [NA]
*MET* SNV in LUAD	39	NGS	0	38	1	0	NA [NA]	100% [NA]
*NRAS* SNV in COAD	5	NGS	0	5	0	0	NA [NA]	100% [NA]
*BRAF* SNV	46	NGS	5	41	0	0	100% [NA]	100% [NA]
*KRAS* SNV	46	NGS	4	42	0	0	100% [NA]	100% [NA]
*ERBB2* CNV	46	NGS	2	44	0	0	100% [NA]	100% [NA]
*FGFR1* CNV	41	NGS	1	40	0	0	100% [NA]	100% [NA]
*FGFR2* CNV	41	NGS	1	40	0	0	100% [NA]	100% [NA]
All SNV	46	NGS	22	157	1	0	95% [76–99]	100% [NA]
All indel	41	NGS	12	29	0	0	100% [NA]	100% [NA]
All CNV	46	NGS	4	122	0	0	100% [NA]	100% [NA]

Abbreviations: CI, confidence interval; CNV, copy number variant; COAD, colorectal adenocarcinoma; FN, false negative; FP, false positive; indel, insertion/deletion; LUAD, lung adenocarcinoma; MSI, microsatellite instability; NPA, negative percent agreement; PPA, positive percent agreement; SNV, short nucleotide variant; TN, true negative; TP, true positive.

In a single LUAD sample, a discordant result was observed. A *MET* exon 14 p.D1028N (chr7:116412043G>A, c.3082G>A, an exon 14 skipping mutation [22, 23]) with a VAF of 22% was detected by the Solo test Driver panel and was missed by the reference panel presumably due to panel design not covering the mutation site.

Test result concordance was further compared with both gold standard methods (PCR, IHC) and the reference panel. In this analysis, MSI status was also included. A total of 63 samples were included in the concordance analysis (41 (65.1%)—LUAD, 20 (31.8%)—COAD, 1 (1.6%)—STAD, and 1 (1.6%)—GI NOS). Overall, 48 (76.2%) samples were considered positive as measured by the gold standard. Of those, 21 were *EGFR*‐positive, 14 were MSI‐positive, 8 were *BRAF*‐positive, 7 were *KRAS*‐positive, 2 were *ERBB2* amplification‐positive, and single samples were *NRAS*, *FGFR1*, or *FGFR2* amplification‐positive. Of note, several samples had several alterations. Diagnostic sensitivity and specificity in relation to MSI detection were 92.86% [95% CI, 66.13%–99.82%] and 100%, respectively; PPV was 100% (no false positive results were observed), NPV was 80.00% [95% CI, 37.70%–96.36%]. In relation to the genetic alteration detection, sensitivity was 100.00% [95% CI, 91.19%–100.00%] and specificity was 92.86% [95% CI, 66.13%–99.82%]; PPV and NPV were 97.56% [95% CI, 85.82%–99.62%] and 100.00% (no false negative results were observed), respectively.

**FIGURE 5:**
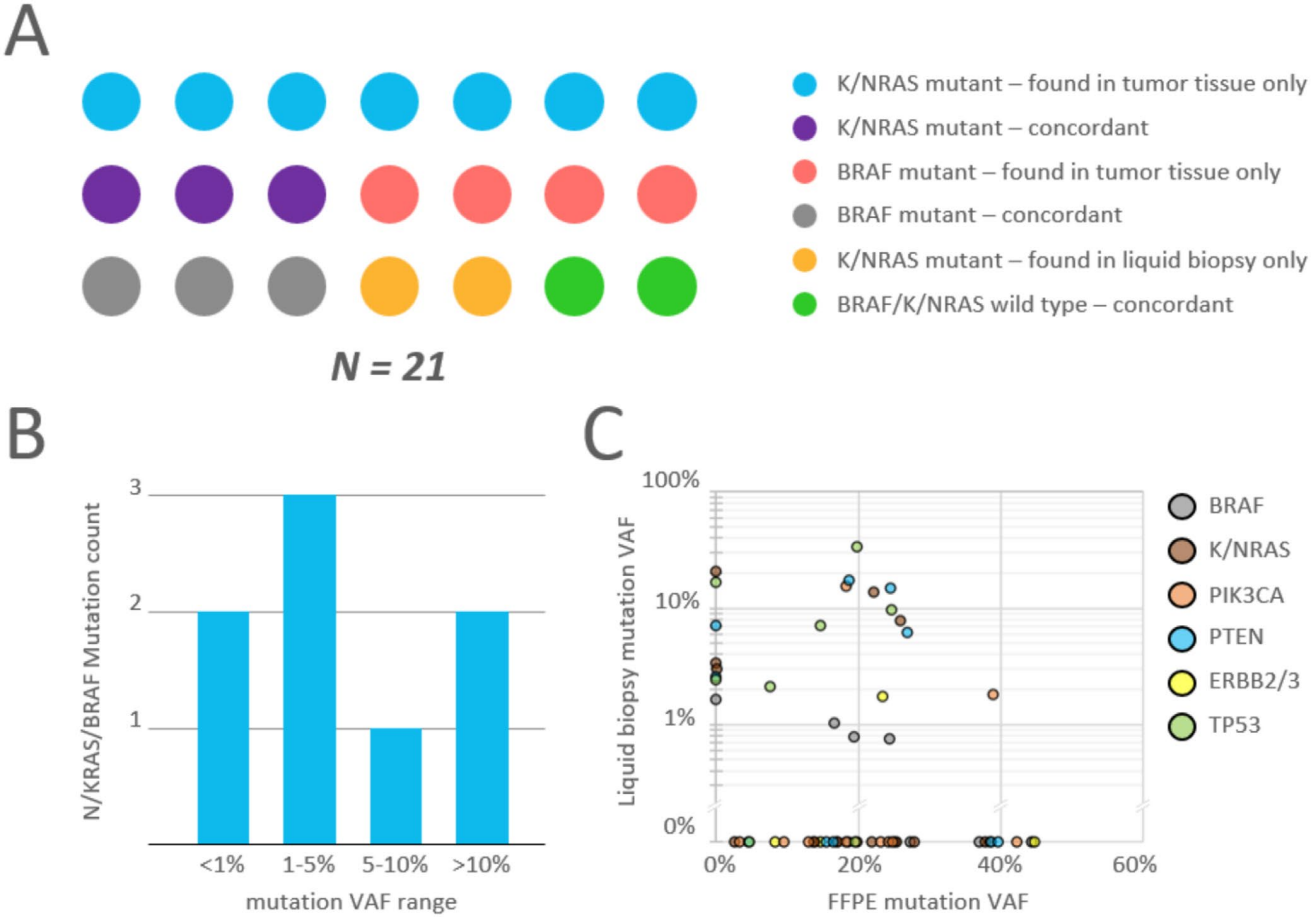
Concordance analysis of paired FFPE and liquid biopsy samples from colorectal cancer patients who were candidates for immunotherapy (MSI‐positive disease). (A) patient population, (B) distribution of VAF of K/NRAS/BRAF mutation identified in liquid biopsy, (C) per‐mutation VAF in liquid biopsy and FFPE samples (VAF of mutations not detected in either sample denoted as 0).

Finally, we performed sequencing of 21 paired tumor‐liquid biopsy (LB) samples from patients with metastatic colorectal cancer who were candidates for immunotherapy (MSI‐positive disease) in order to assess the performance of the panel on LB samples (Figure 5). LB samples were collected before immunotherapy treatment. Excluding potentially passenger somatic mutations, a total of 61 unique alleles were identified. Of these, 58 (95%) were successfully detected in a tumor sample and 20 (32%) were successfully detected in a paired LB sample. A total of 19 patients had detectable alterations in *NRAS*/*KRAS*/*BRAF* genes. Of these, two patients were found to be *K*/*NRAS*‐positive exclusively based on LB analysis results. Sensitivity and specificity in relation to the detection of variants in *NRAS*/*KRAS*/*BRAF* using LB compared to FFPE were 33% [95% CI, 13%–60%] and 95% [95% CI, 85%–99%], respectively. Sensitivity/specificity in relation to the detection of *BRAF*‐positive and *RAS*‐positive status using LB compared to FFPE were 30%/81% and 38%/100%, respectively. A total of 2 *N*/*KRAS*/*BRAF* mutations (25%) were detected with a variant allele frequency of 1% or lower, demonstrating high analytical sensitivity in the context of decent diagnostic sensitivity and suggesting a low presence of somatic mutations in the bloodstream before immunotherapy treatment in this patient population.

## 4 Discussion

NGS is becoming a method of choice for testing for multiple genomic alterations in various cancer types. Among others, the main advantage of NGS is the simultaneous testing for multiple alterations at once using only one sample. In this article, we describe the validation and performance of an in‐house developed amplicon‐based NGS panel for the detection of SNV, CNV, and indel variants in the main genes with a high rate of potentially targetable driver mutations across tumor types. Additionally, our panel includes loci for MSI detection, which is relevant for all solid tumors due to the tumor‐agnostic indications of several immune checkpoint inhibitors [24, 25]. NGS has been described as a promising alternative to conventional testing via 5‐loci PCR and 4‐antibody IHC [26].

PPV and NPV for SNV, indel, and CNV in the clinically relevant cancer‐associated genes included in the Solo test Driver panel were comparable to the PPV and NPV reported for FDA‐approved companion diagnostic NGS‐based panels, as well as other widely used test systems in clinical practice. For instance, PPA and its corresponding 95% CI for the FoundationOne CDx for the detection of substitutions is reported to be 96.6% [95% CI, 95.4%–97.6%] and NPA at 99.9% [95% CI, 99.8%–99.9%], whereas our test system resulted in a 95.6% [95% CI, 75.7%–99.3%] PPA and 100% (NA) NPA for SNV (Table 1). In terms of indel detection, our panel outperformed FoundationOne CDx, with PPA and NPA for FoundationOne CDx at 83.4% [95% CI, 77.6%–88.2%] and 99.9% [95% CI, 99.9%–99.9%]. However, this could be attributed to the larger size of the validation cohort used for FoundationOne CDx validation studies [27]. Furthermore, the only indels detected in our validation cohort were *EGFR* exon 19 indels. Sensitivity and specificity of SNV and indel detection were also comparable with the Oncomine Focus panel. The reported sensitivity and specificity for this assay are > 99% for SNVs and > 99% for indels, respectively [28]. Performance of the Solo test Driver panel for MSI detection was also similar to the FoundationOne CDx (PPA 100% [95% CI, 47.8%–100%] and NPA 99.5% [95% CI, 96.6%–99.98%]) [27]. High analytical sensitivity was also shown when comparing expected and obtained VAFs with an r‐squared correlation coefficient of 0.992.

Systemic false negative results have been reported when using NGS‐based methods for the detection of *MET* exon 14 skipping (METex14) mutations. METex14 mutations occur primarily in non‐small cell lung cancer and can be targeted by small molecule receptor tyrosine kinase inhibitors such as capmatinib, tepotinib, and crizotinib, a non‐specific multikinase inhibitor [29, 30, 31]. Thus, the development of accurate and reliable methods of METex14 detection is crucial for the optimal management of lung cancer patients. However, Amplicon‐based methods are most likely to underdetect METex14 when compared to hybrid capture‐based panels, with the potential to miss up to 37% of METex14 mutations, as reported for the commercially available targeted NGS panels [32]. However, the optimization of amplicon‐based panels can result in improved rates of METex14 detection [33, 34]. RNA‐based approaches have been reported to yield a higher METex14 identification rate when compared to amplicon‐based DNA testing; however, a major of RNA‐based testing is the quality of RNA, which can oftentimes be suboptimal [35, 36]. Although METex14 skipping mutations were first identified using whole exome sequencing (WES) [37], WES is also unreliable in detecting METex14 skipping mutations and is not suitable for clinical settings due to high cost and various methodological obstacles [38, 39]. Transcriptome sequencing, in turn, represents a promising technology for METex14 skipping mutation detection [40]. Another factor worth considering is the complexity of the interpretation of the novel METex14 variants [41]. The NGS panel used as a gold standard for the concordance analysis in our study failed to identify a clinically significant *MET exon 14* p.D1028N variant that was detected in a lung cancer sample by the Solo test Driver panel. This alteration has been identified as one of the most commonly observed METex14 variants [22]. Even though only one sample tested in the course of our validation study was METex14‐positive, the technical potential of the Solo test Driver panel to detect METex14 alterations has been demonstrated.

Ensuring the adequacy of test system performance is a critical responsibility of the manufacturer. We performed an analysis of the panel's quality metrics using additional 58 FFPE, 37 cfDNA, and 25 WB samples. We found that no more than 1.5% of all of the amplicons were systematically dropped out using FFPE and cfDNA. Nonetheless, due to the intersection of genomic regions covered by the panel's amplicons, none of the hotspots were missed. Single‐site sensitivity was 100% for 99.42% of the most clinically relevant alterations (ESCAT I‐III). The downsampling experiments demonstrated that the coverage of clinically actionable variants, including the variants in the critical cancer genes, was consistent with the one recommended by community guidelines [42].

In conclusion, the Solo test Driver panel offers a comprehensive solution for genomic profiling of solid tumors. It demonstrates sensitivity and specificity, even at low coverage depths with high reproducibility and repeatability, making it a reliable alternative to conventional methods. The panel's ability to detect a wide range of genomic alterations, including SNVs, indels, CNVs, and MSI, combined with tailored software for data analysis, makes it a valuable tool to streamline and standardize companion diagnostics of a wide range of solid tumors.

## Author Contributions

**Maxim Ivanov** and **Vladislav Mileyko:** conceptualization. **Maxim Ivanov, Alexandra Lebedeva, Ekaterina Belova, Egor Veselovsky**, and **Vladislav Mileyko:** methodology. **Tatiana Grigoreva, Tatyana Lisitsa, Alexey Barinov, Vladislav Nikulin**, and **Laima Belyaeva:** investigation. **Maxim Ivanov, Alexandra Lebedeva, Ekaterina Belova, Egor Veselovsky, Alexandra Kavun, Anastasiia Taraskina, Vladislav Nikulin, Tatyana Lisitsa**, and **Alexey Barinov:** formal analysis. **Olesya Kuznetsova, Daria Kravchuk, Natalia Pospekhova, Irina Demidova, Alexey Barinov**, and **Saida Aliyarova:** resources. **Maxim Ivanov, Alexandra Lebedeva, Ekaterina Belova, Egor Veselovsky, Alexandra Kavun, Ekaterina Khomenko:** writing – original draft. **Ekaterina Belova, Alexey Tryakin, Mikhail Fedyanin, Irina Demidova, Anna Stroganova**, and **Vladislav Mileyko:** writing – review and editing. **Maxim Ivanov:** funding acquisition.

## Ethics Statement

Study was approved by Sechenov University IRB, declaring a waiver of informed consent.

## Conflicts of Interest

Maxim Ivanov, Alexandra Lebedeva, Ekaterina Belova, Tatiana Grigoreva, Alexandra Kavun, Anastasiia Taraskina, Olesya Kuznetsova, Saida Aliyarova, Ekaterina Khomenko, and Vladislav Mileyko are currently employed by OncoAtlas LLC. Egor Veselovsky ended employment at OncoAtlas LLC in the past 24 months. Other co‐authors have no conflicts of interest to disclose.

## Supporting information


**Table S1.** Genes included in the panel and their clinical (predictive, prognostic, and/or diagnostic) significance. Tumor types are abbreviated according to OncoTree [6]. Levels of evidence (LOE) are listed according to ESCAT [7]. Only the highest LOEs are given. AD, antibody drug conjugates; AODG, anaplastic oligodendroglioma; ASTR, astrocytoma; BLCA, bladder urothelial carcinoma; BRCA, invasive breast carcinoma; CHOL, cholangiocarcinoma; COAD, colon adenocarcinoma; COADREAD, colorectal adenocarcinoma; DIFG, diffuse glioma; GB, glioblastoma; GIST, gastrointestinal stromal tumor; HNSC, head and neck squamous cell carcinoma; ICI, immune checkpoint inhibitors; LUAD, lung adenocarcinoma; MAb, monoclonal antibodies; PAAD, pancreatic adenocarcinoma; PRAD, prostate adenocarcinoma; R, resistance; SERD, selective estrogen receptor degrader; SERM, selective estrogen receptor modulators; SKCM, cutaneous melanoma; STAD, stomach adenocarcinoma; THYROID, thyroid cancer; TKI, tyrosine kinase inhibitors.

## Data Availability

Data will be made available from the corresponding author on reasonable request.
