# Supplementary material for: Analytical and Clinical Validation of Solo‐Test Driver: A Targeted Amplicon‐Based NGS Test‐System for FFPE and cfDNA Analysis in Clinical Oncology Setting
Source: J Clin Lab Anal. 2025 Mar 8;39(6):e70008. doi: 10.1002/jcla.70008 (PMC11937170; doi:10.1002/jcla.70008)
Supplement: Supplementary file 1 — Table S1. Genes included in the panel and their clinical (predictive, prognostic, and/or diagnostic) significance. Tumor types are abbreviated according to OncoTree [6]. Levels of evidence (LOE) are listed according to ESCAT [7]. Only the highest LOEs are given. AD, antibody drug conjugates; AODG, anaplastic oligodendroglioma; ASTR, astrocytoma; BLCA, bladder urothelial carcinoma; BRCA, invasive breast carcinoma; CHOL, cholangiocarcinoma; COAD, colon adenocarcinoma; COADREAD, colorectal adenocarcinoma; DIFG, diffuse glioma; GB, glioblastoma; GIST, gastrointestinal stromal tumor; HNSC, head and neck squamous cell carcinoma; ICI, immune checkpoint inhibitors; LUAD, lung adenocarcinoma; MAb, monoclonal antibodies; PAAD, pancreatic adenocarcinoma; PRAD, prostate adenocarcinoma; R, resistance; SERD, selective estrogen receptor degrader; SERM, selective estrogen receptor modulators; SKCM, cutaneous melanoma; STAD, stomach adenocarcinoma; THYROID, thyroid cancer; TKI, tyrosine kinase inhibitors. [file JCLA-39-e70008-s001.docx]

Supplementary Table 1. Genes included in the panel and their clinical (predictive, prognostic, and/or diagnostic) significance. Tumor types are abbreviated according to OncoTree [6]. Levels of evidence (LOE) are listed according to ESCAT [7]. Only the highest LOEs are given. R - resistance, TKI - tyrosine kinase inhibitors, MAb - monoclonal antibodies, AD - antibody drug conjugates, ICI - immune checkpoint inhibitors, SERD - selective estrogen receptor degrader, SERM - selective estrogen receptor modulators, BRCA - invasive breast carcinoma, PRAD - prostate adenocarcinoma, STAD - stomach adenocarcinoma, LUAD - lung adenocarcinoma, SKCM - cutaneous melanoma, THYROID - thyroid cancer, COADREAD - colorectal adenocarcinoma, GB - glioblastoma, COAD - colon adenocarcinoma, DIFG - diffuse glioma, HNSC - head and neck squamous cell carcinoma, BLCA - bladder urothelial carcinoma, CHOL - cholangiocarcinoma, AODG - anaplastic oligodendroglioma, ASTR - astrocytoma, GIST - gastrointestinal stromal tumor, PAAD - pancreatic adenocarcinoma.

| Gene | Drug class / other clinical significance | Tumor type(s) | ESCAT LOE / Resistance |
| --- | --- | --- | --- |
| AKT1 | AKT inhibitors | BRCA and other solid tumors | I |
| AKT2 | AKT inhibitors | BRCA, PRAD, STAD, and other solid tumors | III |
| AKT3 | AKT inhibitors | BRCA, PRAD, STAD, and other solid tumors | III |
| ALK | ALK TKI | LUAD | I, R |
| ARAF | MEK inhibitors | Solid tumors | III |
| BRAF | BRAF inhibitors  BRAF inhibitors + MEK inhibitors  MEK inhibitors  BRAF inhibitors + anti-EGFR MAb  BRAF inhibitors + MEK inhibitors + anti-EGFR MAb  Anti-EGFR MAb  Diagnostic | SKCM, LUAD, THYROID, COADREAD, BRAIN, and other solid tumors | I, R |
| EGFR | EGFR TKI  EGFR TKI + anti-VEGFR MAb EGFR/MET ADC | LUAD, GB, COAD | I, R |
| ERBB2 | Anti-ERBB2 MAb  ERBB2/chemotherapy ADC  ERBB2 TKI  ERBB2 TKI + Anti-ERBB2 MAb  pan-ERBB TKI  Anti-ERBB2 MAb + ICI | BRCA, LUAD, COAD, STAD, and other solid tumors | I |
| ERBB3 | pan-ERBB TKI | BRCA, STAD, and other solid tumors | II |
| ERBB4 | pan-ERBB TKI | Solid tumors | II |
| ESR1 | SERD  SERM  Aromatase inhibitors | BRCA | I, R |
| FGFR1 | FGFR TKI | BRCA, STAD, LUAD, and other solid tumors | III |
| H3C2 | Prognostic, diagnostic | DIFG | N/A |
| H3C3 | Prognostic, diagnostic | DIFG | N/A |
| HRAS | Farnesyltransferase inhibitors | HNSC, BLCA | II |
| IDH1 | IDH1 inhibitors  Prognostic, diagnostic | CHOL, AODG, ASTR | I |
| IDH2 | IDH2 inhibitors  Prognostic, diagnostic | AODG, ASTR | III |
| KIT | KIT TKI | GIST, SKCM | I, R |
| KRAS | KRAS G12C inhibitors  EGFR MAb | LUAD, PAAD, COAD, and other solid tumors | I, R |
| MET | MET TKI | LUAD, STAD | I |
| NRAS | MEK inhibitors  Anti-EGFR MAb | SKCM, COAD | II, R |
| PDGFRA | PDGFR TKI  Diagnostic | GIST, DIFG | I, R |
| PIK3CA | PI3K inhibitors  AKT inhibitors | BRCA and other solid tumors | I |
| PTEN | AKT inhibitors  Diagnostic | BRCA and other solid tumors | I |
| RAC1 | RAF inhibitors  MEK inhibitors | SKCM | R |
| RAF1 | MEK inhibitors | Solid tumors | IV |
| RIT1 | MEK inhibitors | Solid tumors | IV |
| ROS1 | ROS1 TKI | LUAD | R |
| STK11 | Prognostic | LUAD | N/A |
| TP53 | Prognostic, diagnostic | Solid tumors | N/A |
